# Adsorptive stripping voltammetric sensor based on Cd zeolitic imidazole framework-67 for electrochemical detection of sarin simulant

**DOI:** 10.1007/s00604-023-06112-3

**Published:** 2024-01-08

**Authors:** Mona Elfiky, Amr M. Beltagi, Osama Abuzalat

**Affiliations:** 1 Department of Chemistry, Faculty of Science, Tanta University, Tanta, Egypt; 2 Department of Chemistry, Faculty of Science, Kafrelsheikh University, Kafrelsheikh, 33516 Egypt; 3 Department of Chemical Engineering, Military Technical College, Cairo, Egypt

**Keywords:** Glassy carbon sensor, Modified electrode, Dimethyl methyl phosphonate, Cd zeolitic imidazole framework-67, Stripping voltammetry, Human serum

## Abstract

A selective and reliable modified glassy carbon sensor, based on a 1.0% Cd zeolitic imidazole framework-67 modified glassy carbon sensor (GCS_2_), has been developed for ultrasensitive detection of dimethyl methyl phosphonate (DMMP) in human biological fluid. The synthesis of porous nanoparticles of Cd zeolitic imidazole framework-67 (Cd ZIF-67) was carried out via the hydrothermal method. The resulting Cd ZIF-67 powder emerges with good crystallinity, a rhombic dodecahedral morphology with particle size in the range 300 ~ 500 nm, and a specific surface area of 1780 m^2^·g^−1^. Furthermore, the fabricated sensor exhibited superior performance in the detection of DMMP with two linearity ranges of 0.02–2.0 nM and 2.0–9.0 nM and a limit of detection (LOD) of 0.06 pM. The fabricated sensor exhibited good reliability, long-term stability, and repeatability, which are favourable attributes for electroanalytical detection. In addition, the fabricated sensor displayed superior performance without significant interference during the assay of DMMP in a biological fluid (human serum sample) within two linearity ranges of 0.1–1.0 nM and 1.0–6.0 nM and a LOD of 0.03 nM.

**Graphical Abstract:**

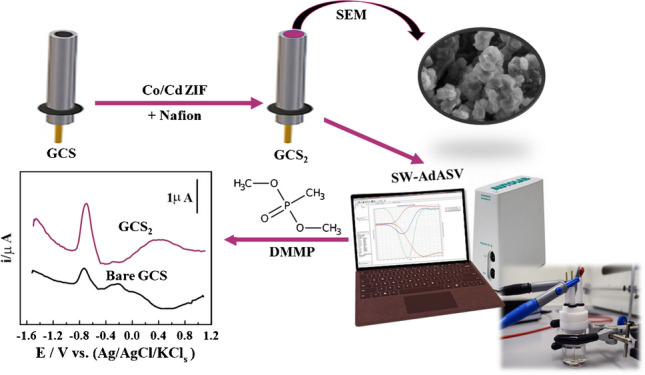

**Supplementary Information:**

The online version contains supplementary material available at 10.1007/s00604-023-06112-3.

## Introduction

Chemical warfare agents, for instance sarin, are very toxic and often fatal chemicals that pose substantial health and safety risks. Dimethyl methyl phosphonate (DMMP) (Scheme.S_1_) has a comparable structure to sarin and is thus commonly used as a chemical warfare agent simulant [1]. Such nerve agents inhibit the acetylcholinesterase enzyme, causing the accumulation of an immoderate level of a neurotransmitter (acetylcholine) at cholinergic synapses [2]. The military threat posed by the use of these materials as chemical warfare agents increases the need for quick, efficient, and selective analytical sensors to detect such materials in different types of biological and environmental fluids [3]. DMMP has reportedly been detected using a variety of analytical techniques, including the use of several types of sensors [4–17]. In recent studies, Dipak et al. [10] and Alev et al. [13] have fabricated gas sensors based on aluminum-doped nickel ferrite nanoflakes and WS_2_-coated quartz crystal microbalance for the detection of DMMP in bulk form at room temperature with LODs of 0.8 µM and 0.04 µM, respectively. In addition, McKenna et al. [16] have successfully used paper spray mass spectroscopy to detect warfare agent products in the urine and blood samples with LODs of 0.1 and 0.23 µM (12.6 and 28.6 ng mL^−1^), respectively, as shown in Table S1. Shaik et al. have successfully fabricated *p*-hexafluoroisopropanol phenyl functionalized graphene as a strong hydrogen bond acidic polymer to interact with DMMP in its vapor state, which is considered one of the hydrogen-bond basic organophosphorus compounds using a quartz crystal microbalance (QCM) sensor [14]. As illustrated in Table S1, most of the reported methods need a thermal analysis step to convert DMMP to its gaseous state before the main analysis process. The cyclic voltammetry technique, which uses a modified gold sensor based on the electro-polymerization of pyrrole, has only been applied by Sharma et al. [11] (Table S1) for the determination of DMMP. Due to its high selectivity and sensitivity (up to picomolar concentration levels), the adsorptive stripping voltammetry technique (AdSV) has devoted great attention to the field of sensing different species in different fluids [18]. To date, there are no detailed reports on the usage of the adsorptive stripping voltammetry technique (AdSV) for the detection of DMMP in bulk and/or biological fluids using modified electrochemical sensors. Metal–organic frameworks (MOFs) [19] are promising new adsorbent frameworks with robust adsorption and well-ordered porosity features [20]. However, the poor conductivity and stability of most MOFs reduce their usability in the field of electrochemical sensing applications. A zeolitic imidazole framework (ZIF) is a special subclass of MOFs [21], which are formed through a self-assembly method via the linking of metal ions including Zn^2+^ or Co^2+^ with organic ligands, including imidazole or imidazole derivatives. ZIFs have great potential in different applications due to their superior features such as thermal/hydrothermal stability of the framework, high specific surface area with well-ordered porosity, and robust adsorption properties [22]. ZIF-67 is a rhombic dodecahedron ZIF with a 3D pore structure [22]. Due to the redox property of Co^2+^, which is accompanied by excellent porosity, and stability properties, ZIF-67 is a superior modifier in sensing applications [22, 23]. To improve the weakness of the conductivity property of ZIF-67 MOFs, preparation of multi-metal ZIF-67 is essential to merge the advantages of each metal ZIF material, which may improve the electrochemical sensing performance [24, 25].

In this work, porous nanoparticles of Cd zeolitic imidazole framework-67 (Cd ZIF-67) were synthesized via the hydrothermal method. Afterward, a selective and reliable modified glassy carbon sensor has been developed, based on 1.0% Cd ZIF-67 (GCS_2_), for ultrasensitive determination of DMMP. In addition, the as-prepared sensor was utilized to estimate DMMP in human serum samples without significant interference from the common biological interferents during the analysis process.

## Experimental part

### *Materials, apparatus, electroanalytical solutions, and the point of zero charges (pH*_*ZPC*_*) measurements*

This part is detailed in the supplementary material section.

### Synthesis of Cd ZIF-67 powder

In a typical synthesis, 0.34 g (1 mM) of cadmium nitrate hexahydrate and 0.291 (1 mM) of cobalt nitrate hexahydrate were dissolved in 20 mL of methanol under stirring to prepare the metal solution. Two grams of triethylamine and 1.64 g of 2-methylimidazole were dissolved in 20 mL of methanol to prepare the ligand solution. The metal solution was then poured into the ligand solution under stirring and continually stirred for 1 h. The mixed solution was then transferred into a teflon-lined autoclave. The autoclave was placed in the oven at 60 °C for 48 h. After the reaction was done, the powder was collected, washed with methanol, and dried under vacuum at room temperature.

### Fabrication of bare and modified sensors

The bare GCSs were cleaned by polishing with a 0.05 μm alumina slurry and sonicated several times with a solution of DDW and ethanol (1:1) for 10 min. The modified GCS (GCS_1_) was carried out as follows: 4.0 mg of Cd ZIF-67 powder was dispersed in 1.0 mL of ethanol and 0.1 mL of a 0.5% nafion solution, followed by ultrasonication for 15 min. Then, 8.0 μL of suspension was dropped on the surface of GCS and allowed to air dry at room temperature. The same procedure was performed utilizing 8.0 mg (1.0%) and 12.0 mg (2.0%) of Cd ZIF-67 powder to fabricate GCS_2_ and GCS_3_.

## Results and discussion

### The charge delocalization of DMMP

The fabrication of super-sensitive and selective electrochemical sensors mainly depends on providing diverse properties, including a low degree of resistivity (*R*_ct_), better surface area, and adsorption, as well as a better electrostatic attraction force between the proposed sensor and the solvated analyte throughout the analysis process. Therefore, the charge delocalization of DMMP was tested and estimated to be − 1.8858431 concerning the density functional theory (DFT) method, as illustrated in Table 1.

**Table 1:** Data of Mulliken atomic charge of DMMP structure

Atom	Mulliken atomic charges	DMMP charge without H atoms	Overall DMMP charge
P	0.7111409	− 1.8858431	0.0
O	− 0.508380		
O	− 0.291168		
O	− 0.443817		
C	− 0.786972		
H	0.270428		
H	0.197125		
H	0.206583		
H	0.283538		
C	− 0.270596		
H	0.144688		
H	0.133786		
H	0.139845		
C	− 0.296051		
H	0.170421		
H	0.170230		
H	0.168931		

### Characterization of Cd ZIF-67 powder

**Fig. 1:**
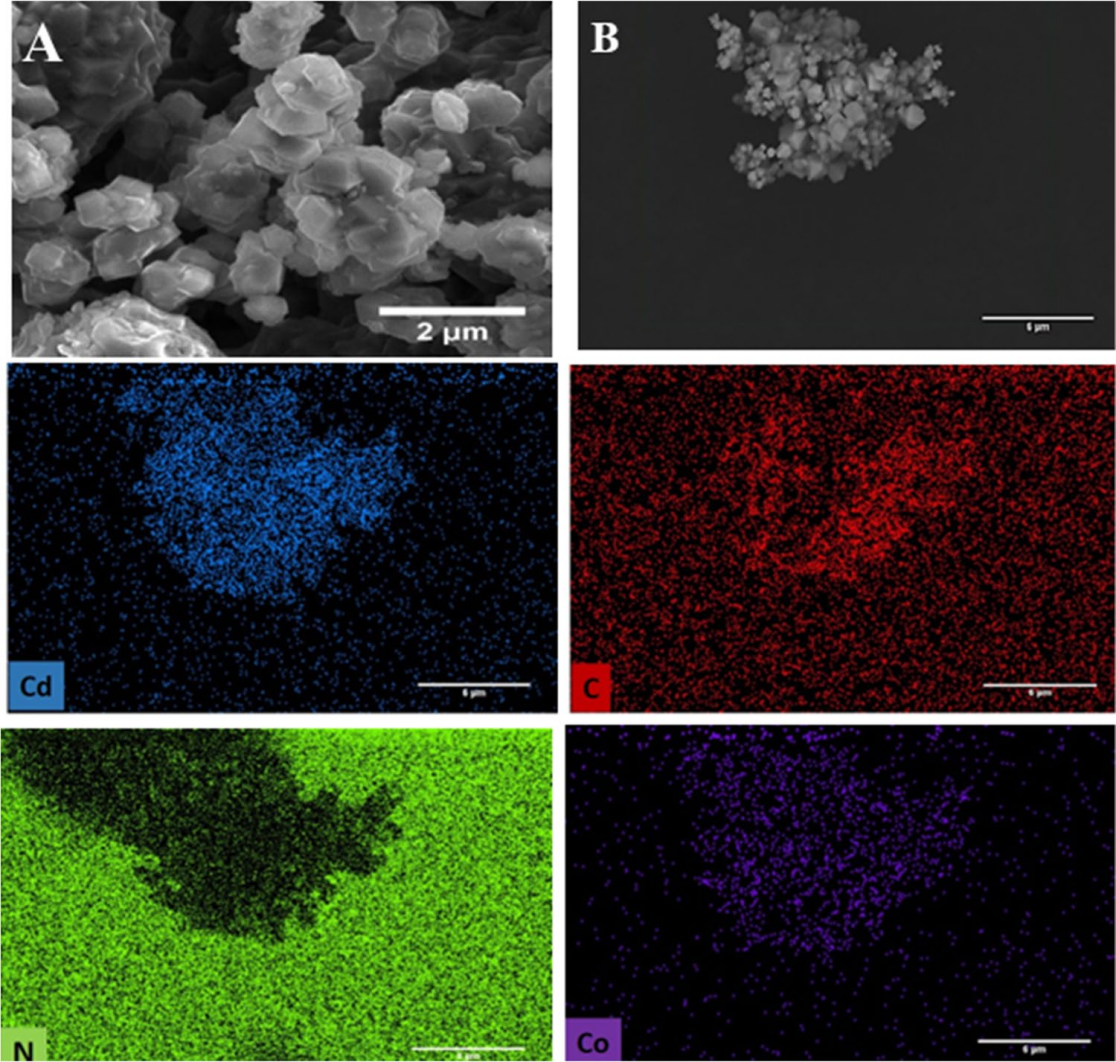
**A** SEM image and **B** SEM image coupled with the EDX elemental mapping distribution of Cd, C, N, and Co elements of Cd ZIF-67

Visual examination reveals that the reaction of cobalt and cadmium nitrate hexahydrate with 2-methylimidazole in methanol yields the pure dark brown crystalline product of Cd ZIF-67. Scanning electron microscopy confirms a rhombic dodecahedral morphology of Cd ZIF-67 and its particle size distribution in the range of 300 ~ 500 nm, as shown in Fig. 1A and Fig. S1. Furthermore, the SEM–EDX demonstrated the elemental mapping distribution of Cd, C, N, and Co elements in the Cd ZIF-67 sample, proving the incorporation and uniform distribution of Cd and Co elements in the sample, as displayed in Fig. 1B.

**Fig. 2:**
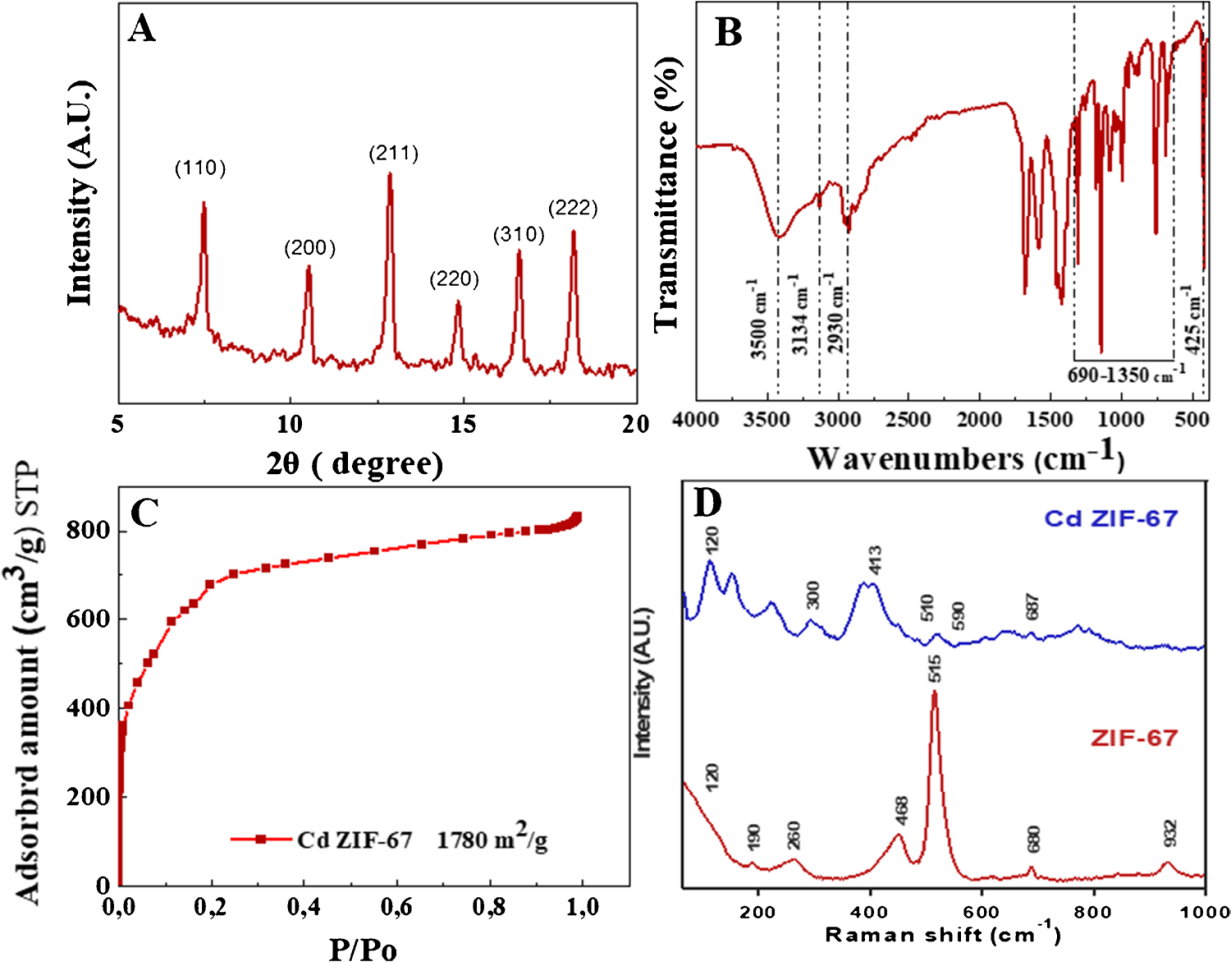
**A** PXRD pattern, **B** FTIR, **C** nitrogen sorption measurements at 77 K, and **D** Raman spectra of Cd ZIF-67

X-ray diffraction patterns in Fig. 2A demonstrate that a pure ZIF-67 topology phase is formed, which showed similar patterns and was in good agreement with the simulated pattern [26, 27]. The comparative intensities and notable peak positions at 2*θ* = 7.3°, 10.35°, 12.7°, 14.8°, 16.4°, and 18° correspond to (110), (200), (211), (220), (310), and (222), respectively. This is in good agreement with previous reports [28, 29]. The values of grain size were determined by Scherrer’s equation [30].

The estimated crystallite size was 83.52 nm. As well, the FT-IR spectra in Fig. 2B further demonstrate the structural analysis of Cd ZIF-67. At higher frequencies, Cd ZIF-67 showed three contributions. The first one is a broad peak between 3500 and 3200 cm^−1^ due to the stretching of the N–H bond of protonated imidazole [31, 32], and the other two are the small peaks present at 3134 and 2925 cm^−1^, which can be ascribed to C–H stretching vibrational modes of the imidazole ring and the methyl group existing in the organic ligand [33]. The peak at 1591 cm^−1^ may be due to the C = N stretch modes, while the peak at 1457 cm^−1^ agrees with the entire ring stretching [32]. Several spectral bands were found in the range of 1350 to 900 cm^−1^ that might be related to the ring’s in-plane bending. The peaks at 760 and 690 cm^−1^ correspond to aromatic sp2 C–H bending [34]. The peak at 425 cm^−1^ corresponds to the Co–N bond [35].

Furthermore, Fig. 2C presents the N_2_-adsorption isotherms Cd ZIF-67, and accordingly, the BET surface area is 1780 m^2^/g. The obvious high surface area could confirm the effective fabrication of highly porous Cd ZIF-67.

In addition, the point of zero charges (*pH*_pzc_) is described as a pH at which the surface charge density of the surface of fabricated molecules is equal to zero [18]. In this work, the *pH*_pzc_ of Cd ZIF-67 was found to be equal to *pH* 7.3, as illustrated in Fig.S_2_. Subsequently, at *pH* ≤ 7.0, the net surface charge of Cd ZIF-67 is positively charged (+ *ve*), whereas at *pH* ≥ 8.0, the net surface charge is negatively charged (− *ve*).

As shown in Fig. 2D, ZIF-67 and Cd ZIF-67 were further studied using Raman spectroscopy. The peaks at 413, 468, 515, and 680 cm^−1^ are attributed to ZIF-67, including the Co–N bond at 413 cm^−1^ and the vibrational mode of the 2-methyl imidazolate ligand at 680 cm^−1^ [36]. The peaks at 468, 515, 607, and 687 cm^−1^ belonged to the four Raman active modes of *E*_g_, *F*^1^_2g_, *F*^2^_2g_, and *A*^1^_g_ for the Co–O bond [37]. Cd ZIF-67 spectrum (Fig. 2D) showed new peaks around 300, 510, and 590 cm^−1^. The peak around 300 cm^−1^ is the 1LO (longitudinal optical) phonon arising from the A1 mode of the Cd–N bond vibration, and the peak at 590 cm^−1^ is caused by the offset of Cd in the Cd ZIF-67 composite [38]. In addition, peaks that can be observed at 120, 413, and 687 cm^−1^ are evidence for the retention of ZIF-67 [36, 38].

XPS survey spectra of Cd ZIF-67 are shown in Fig.S_3_(A). The spectrum shows that the incorporated elements in the structure are C, N, Co, and Cd, and their atomic abundance percentages are 61.2, 28.3, 4.1, and 6.4%, respectively. The Cd ZIF-67 N 1 s core-level spectra are illustrated in Fig.S_3_(B). They are separated into several peaks, depending on the nitrogen environment. The deconvolutions were divided into four peaks. The pyridinic nitrogen atoms may be responsible for the strongest N 1 s peaks at 398.8 eV [39]. This peak denotes the successful coordination through nitrogen-sp2 bonding with metal ions due to the small difference in binding energies between the metal-coordinated N and pyridinic N [40]. It further specifies that most methyl imidazole reactions should result in methyl imidazolate. The deconvoluted C 1 s spectra are shown in Fig.S_3_(C). There are three major peaks at 284.57, 284.99, and 288.15 eV. These peaks may be attributed to the C–C/C = C [41–43], C = N [44–46], and C–N [43, 46, 47], respectively. The Co 2p deconvoluted spectra are shown in Fig.S_3_(D). There are two primary peaks visible. These peaks are indexed to Co 2p3/2 and 2p1/2 spin–orbit splitting at 780.9516 and 797.24 eV. The deconvoluting cobalt Co 2p3/2 and Co 2p1/2 spectra present two main peaks under each one along with their significant corresponding satellite peaks. The peaks at 782.94, 786.5, and 789.44 eV correspond to the unsaturated Co species in Co–N coordination within the framework [48]. Furthermore, Fig.S_3_(D) shows the XPS spectra of Cd incorporated in Cd ZIF-67, showing two main peaks at 412.37 and 405.21 eV, which are recognized as Cd 3d3/2 and Cd 3d5/2, respectively, representing the Cd^2+^ oxidation state [49]. The spin–orbit interaction with Cd(3d5/2) and Cd(3d3/2) produced the fine doublet at energies of 406.51 and 411.85 eV, respectively [50].

### Electrochemical characterization of as-prepared sensors

Previous studies [51, 52] indicated that metal nanoparticles co-formulated with modified electrode materials rarely undergo oxidation in aqueous buffer solutions [52], except [53, 54] when they are exposed to a strongly acidic medium of 0.5 M HCl (pH 0.3) [53]. The electrochemical behavior of the GCS_2_ was first studied by cyclic voltammetry to prove the incorporation of Cd in ZIF-67. Cyclic voltammograms for both BGCS and GCS_2_ were recorded in a 0.5 M HCl solution (Fig.S_4_). The electrochemical response for BGCS (red line, curve a) shows no redox processes; however, the GCS_2_ (blue line, curve b) showed a characteristic oxidation process at − 898 mV and a reduction process at − 982 mV in the reverse scan for the Cd redox process.

#### Electroactive surface area and resistive properties

ZIF-67 usually has a positive surface charge [55, 56]. Nafion, a negatively charged perfluorinated polymer, can bind to positively charged ZIF-67 and form a stable porous film on electrode surfaces. This agrees with the results of the point of zero charges previously mentioned. As previously reported, contacting ZIF-67 with water could result in a *pH* increase, which might be due to the protonation effect of imidazolate groups (mim) exposed at the outer surface of the ZIF-67 particles [57], according to the equation:

$${mim}^{-}+{H}_{2}O\iff Hmim+{OH}^{-}$$

The interactions between anionic species and ZIF-67, which has a positive surface charge, were made easier by the solution’s increased *pH*. Furthermore, the uncoordinated Co^2+^ sites at the outer surface of ZIF-67 were deemed to bond with hydroxide derived from water in an aqueous solution [58]. This means that ZIF-67 could adsorb anionic [Fe(CN)_6_]^3−/4−^species through electrostatic interactions.

**Fig. 3:**
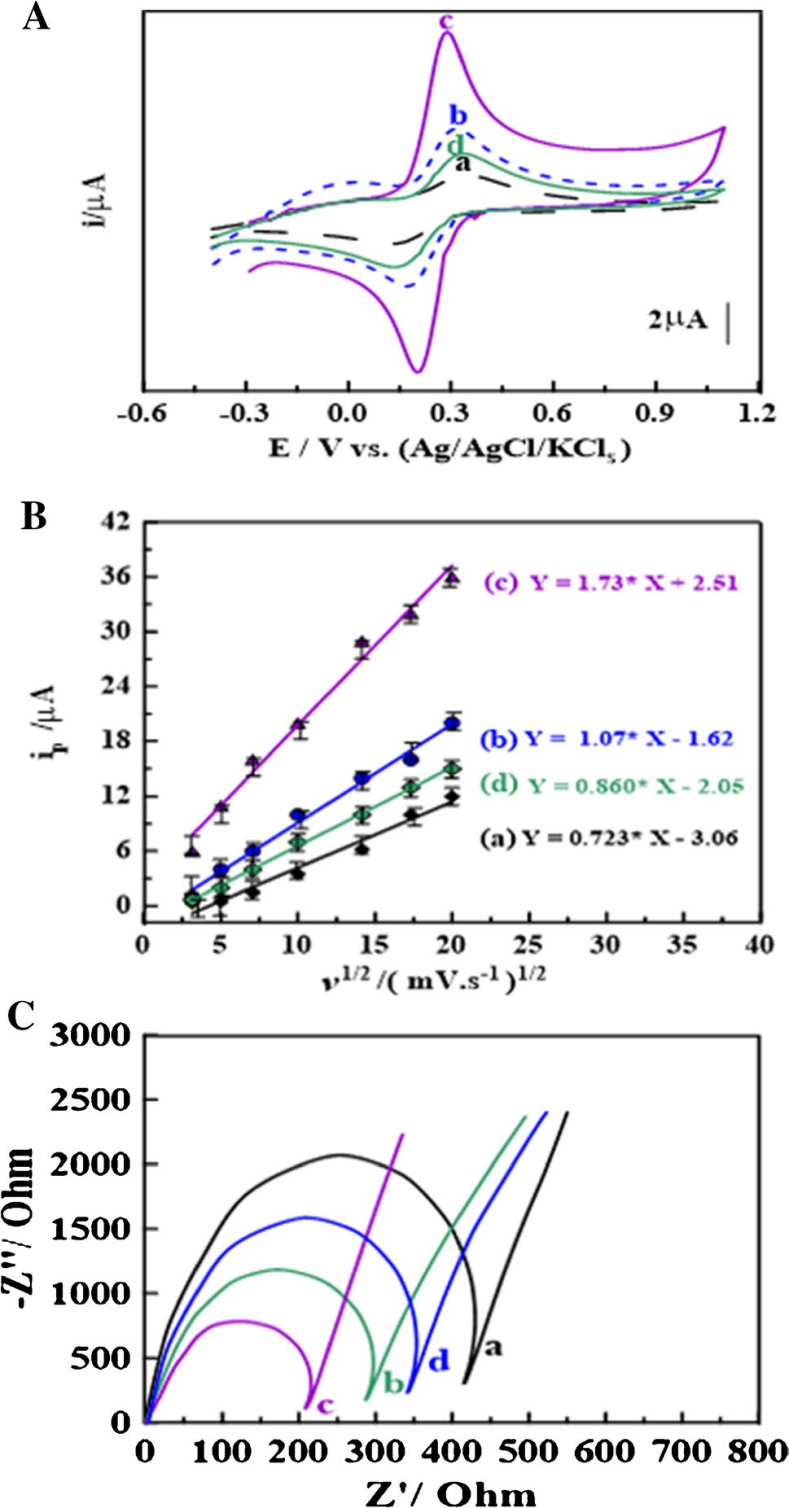
**A** CVs of 10 mM of K_3_[Fe(CN_6_)] in 0.1 M of KCl (*v* = 100 mV·s^−1^), and **B** plot of *i*_p_ vs. *v*^1/2^ relating CVs at *v* ≈ 10–400 mV·s.^−1^ using (a) BGCS and (b) GCS_1_, (c) GCS_2_, and (d) GCS_3_ sensors (*n* = 3). **C** Nyquist plots of 10.0 mM K_3_[Fe(CN_6_)] in 0.1 M of KCl utilizing (a) BGCS, (b) GCS_1_, (c) GCS_2_, and (d) GCS_3_ sensors (*n* = 3)

The electroactive surface area of bare and modified stripping voltammetric glassy carbon sensors was measured to provide more information about the electrochemical properties of the surface of BGCS and as-prepared sensors. Figure 3A demonstrates the CV of 10 mM K_3_[Fe(CN_6_)] in 0.1 M of KCl as a redox probe cell system using BGCS and GCS_1-3_ sensors (scan rate (*ν*) of 100 mV·s^−1^). The CV voltammograms demonstrate a well-defined redox peak, owing to the reversible electron transfer rate of [Fe(CN)_6_]^3−/4^ compared to unmodified GCS. Moreover, the redox peaks display Δ*E*_p_ values almost equal to 140, 100, and 150 mV for the GCS_1_, GCS_2_, and GCS_3_ sensors, respectively, compared to the BGCS (Δ*E*_p_ = 210 mV).

Notably, the amount of charges on the as-prepared sensors’ surface causes an acceleration of the rate of electron transfer of [Fe(CN)_6_]^3−/4−^on the as-prepared sensors’ surface, which accounts for the Δ*E*_p_ values decreasing for some of the as-prepared sensors. Furthermore, [Fe(CN)_6_]^3−/4−^ had its highest redox peak current intensity at the GCS_2_ sensor, which may be due to the large surface area of Cd ZIF-67, and the good conductivity of co-mixed metal ions (Co and Cd).

By using the Randles–Sevcik equation [59], the electroactive surface area (*A*_surface_) of all as-prepared sensors was evaluated from *i*_p_ versus *v*^1/2^ plots relating the CV of 10 mM of K_4_[Fe(CN_6_)] in 0.1 M of KCl at *v* ≈ 20–400 mV∙s^−1^ (Fig.S_5_ and Fig. 3B) according to the following equation:

*i*_p_ = (2.69 × 105) *n*^3/2^*A*_surface_ *D*^1/2^* v*.^1/2^

where *n* is the amount of e^.^ in the electrochemical process, *D* is the diffusion coefficient (7.6 µcm^2^·s^−1^), *A*_surface_ is the electroactive surface area of the as-prepared sensor, and *C* is the concentration of K_3_[Fe(CN)_6_]. The values of the electroactive area of BGCS and GCS_1-3_ sensors were calculated to be 0.056, 0.13, 0.21, and 0.11 cm^2^, respectively. It is noteworthy that the GCS_2_ sensor has the highest *A*_surface_ value, with a fourfold increase compared to the BGCS.

Furthermore, the charge transfer resistance (*R*_ct_) at the interface of BGCS and as-prepared sensors was accounted for via electrochemical impedance spectroscopy (EIS) [60]. Nyquist plots for BGCS and GCS_1-3_ sensors were measured in 0.1 M of KCl containing 10.0 mM K_3_[Fe(CN_6_)] to provide more details about the performance of the sensing mechanism of all as-prepared sensors (Fig. 3C). *R*_ct_ values were evaluated to be 420 ± 1.53, 290 ± 2.51, 200 ± 2.08, and 330 ± 2.08 Ω for the BGCS and GCS_1-3_ sensors, respectively. The GCS_2_ exhibited a development in the rate of electron transfer with low resistivity compared to the BGCS and all other as-prepared sensors.

#### Electrochemical behavior and sensor reaction mechanism of DMMP

**Scheme 1:**
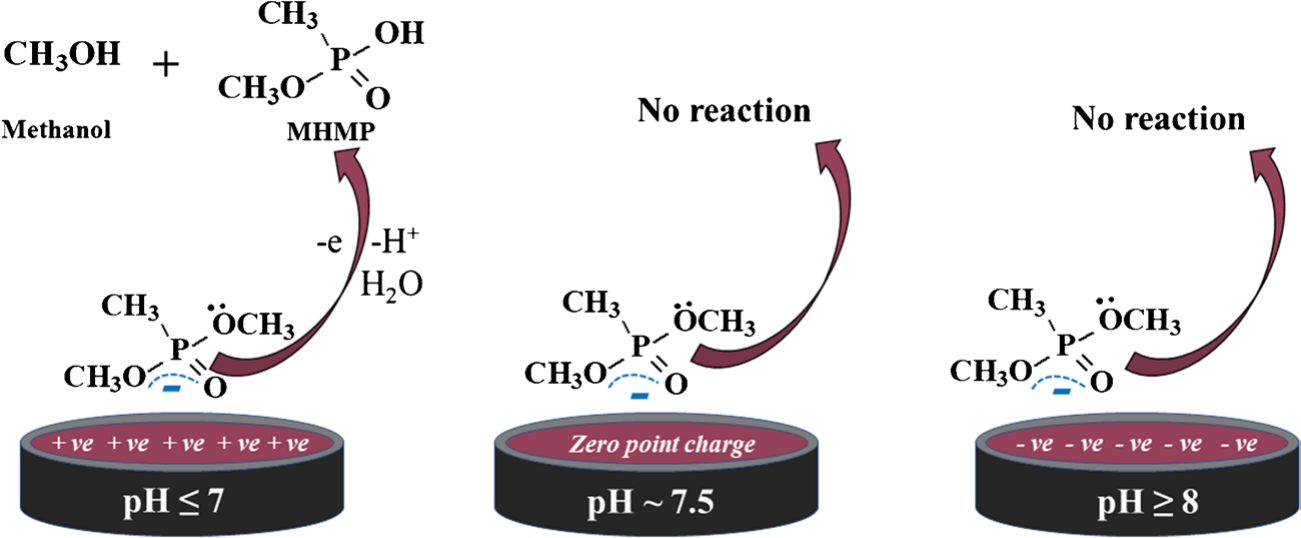
The proposed mechanism for the oxidation of DMMP

To clarify the electrochemical behavior of DMMP, cyclic voltammograms (CVs) of 1.0 μM DMMP were measured in B-R universal buffer of different pH values upon the surface of GCS_2_ at scan rate (*v* = 100 mV·s^−1^). As displayed in Fig.S_6_, the CV of DMMP in *pH* 4 displayed the main oxidation peak at peak potential (*E*_p_) ≈ − 0.75 V, which could be related to the selective oxidation of the phosphonate group into phosphate [61, 62]. The voltammogram did not show the oxidation/reduction couple for Cd in the sensor material, which means that Cd nanoparticles in the sensor material are electrochemically inert in the investigated B-R buffer solutions. No anodic peak was observed in the reverse scan upon increasing the scan rate from 10 to 500 mV/s suggesting that the oxidation reaction of DMMP can be chemically irreversible electron transfer for the scan rates being used, in which the redox event is followed by a chemical reaction (EC mechanism) [63]. On the other side, it was observed that *E*_p_ shifts linearly in negative direction with an increase in *pH*, following the equation: *E*_p_ (V) = (− 0.067 ± 0.0022) *pH* + (0.45 ± 0.031) (*R*^2^ = 0.998 and *n* = 6) (*pH* of 2.0–7.0), as shown in Fig. S_7_(B), which indicates the involvement of protons in the electrochemical oxidation of DMMP at the surface of GCS_2_. Generally, the number of protons participating in the electrochemical reaction was calculated for the reversible or quasi-reversible electrode reaction process [64]. This slope value of 0.067 V/*pH* was close to the Nernst slope of 0.059 V/*pH* at 25 °C. This result indicated that an equal number of protons and electrons took part in the oxidation process of DMMP [65]. Furthermore, the transfer coefficient (*α*) was evaluated to be 0.73 while assuming the number of electrons (*n*_a_) is equal to 1 from the slope value of the linear plot of *E*_p_ vs. ln *v* of 1.0 μM DMMP in B-R universal buffer (*pH* 4) using GCS_2_ (Fig.S_7_(C)), which is expressed by the following equation [66]: *E*_p_ (V) = (0.035 ± 0.0037) ln *v* (mV∙s^−1^) + (0.880 ± 0.045) (*R*^2^ ≈ 0.984 and *n* = 8). As for a completely irreversible and reversible electrode reaction, *α* can be taken as 0.5 and 1.0, respectively [67]; the electrode reaction can be considered a quasi-reversible reaction for the scan rates being considered (10–500 mV/s). Further, [Δ*E* = *E*_P_ − *E*_P/2_] of 55 to 60 mV suggested also the quasi-reversible electrode process. For such a quasi-reversible system [68, 69], [*E*_P_ − *E*_P/2_] = 1.857 *RT*/(*αn*_a_*F*), values of *αn*_a_ (0.80–0.87) were also calculated at various *pH* values. These results confirm the participation of an equal number of protons (*p*) and electrontransfer (*n*_a_), which is assumed to be 1 in the oxidation mechanism [70], as demonstrated in Scheme 1. As well, the linear plot of Log *i*_p_ vs. Log *ν* in a B-R buffer *pH* 4 was estimated to investigate the adsorption-controlled process of the DMMP upon the surface of GCS_2_, which is expressed by the following equation: Log *i*_p/μA_ = (1.023 ± 0.004) Log *ν*_/mV·s_^−1^ − (1.84 ± 0.033) (*n* = 8 and *R*^2^ = 0.998), as displayed in Fig.S_7_(D). The obtained slope value suggests that the oxidation process of the DMMP on the surface of GCS_2_ is controlled by the adsorption process [18].

The anodic/forward charge transfer may be followed by a homogenous irreversible chemical reaction [63, 71]. The product of electron transfer fromthe oxidation process has been chemically consumed in the formation of the follow-up product and is therefore not available for reduction at such a low scan rate (i.e., at this time-scale of the experiment, the backward electron transfer reaction could not take place, leaving the forward reaction dominant) [63, 71]. Adsorption effects may also contribute to the absence of the peak in the reverse direction [72]. Samin et al. observed similar results [72] which successfully captured the physics with a model taking into account diffusion, kinetics, adsorption effects, and morphology changes on the electrode surface or the electron transfer step.

As suggested in mechanism (Scheme 1), the methoxy group (O–CH_3_) and phosphoryl oxygen (P = O) of DMMP were linked with the surface of GCS_2_ with an electrostatic attraction force depending on the Lewis acid–base interactions [73]. The methoxy group (O–CH_3_) is first cleaved and protonated to generate methanol (CH_3_OH) as a by-product, accompanied by the formation of methyl hydrogen methyl phosphonate (MHMP) molecules [74, 75]. As shown in Fig.S_7_(A), the *i*_p_ vs. *pH* plot confirmed that the highest *i*_p_ was obtained at *pH* 4, followed by a gradual decrease with increasing *pH* until complete disappearance at *pH* ˃ 8. This electrochemical performance may arise from the electrostatic repulsion between DMMP and GCS_2_, which can be further explained by the following reasons: As stated previously, the charge delocalization throughout the solvated DMMP was measured and displayed with a − *ve* charge (− 1.8858431) using the Gaussian 09 program package. At *pH* ≤ 3.0, the magnitude of *i*_p_ is very small, owing to the repulsion force between the strong protonated form of DMMP molecules (+ *νe*) in the acidic medium (acid–base dissociation constant (*pK*_a_ ~ 2.37 [76])) and the positively charged surface (+ *νe*) of GCS_2_ (*pH*_zpc_ ~ 7.3; Fig.S_2_), as shown in Scheme 1. Thus, the mechanism of the reaction mainly depends on the electrostatic attraction force accompanied by the adsorption characteristics between the DMMP molecules and the surface of GCS_2_, which occur easily at *pH* 4. At *pH* ≥ 8, the *i*_p_ is completely disappeared, due to the occupancy of the surface of DMMP and GCS_2_ with a lone pair of electrons upon methoxy groups (− *νe*) and imidazole groups (− *νe*), which hinders the electrostatic attraction between the DMMP molecules and the surface of GCS_2_.

#### The adsorption behavior and the preliminary stripping voltammetry test of as-prepared sensors

The preliminary anodic stripping voltammetric scan of 2.0 pM of DMMP in the BR buffer *pH* 4 (Fig.S_8_(A)) was recorded utilizing BGCS, GCS_1_, GCS_2_, and GCS_3_ sensors at an accumulation potential (*E*_acc_) of − 1.5 V (versus Ag/AgCl/KCl_s_) using the square-wave adsorptive anodic stripping voltammetry technique (SW-AdASV) at *t*_acc_ = 30 s, *a* = 25 mV, *f* = 100 Hz, and Δ*E*_s_ = 10 mV. Notably, the *i*_p_ of GCS_2_ displayed a greater affinity for DMMP with about a threefold increase concerning the bare CPS, which may arise from the improvements in the electroactive surface area features of the utilized modifier material.

In this context, the adsorption behavior of the DMMP on the surface of the BGCS (inset; Fig.S_8_(B)) and GCS_2_ (Fig.S_8_(B)) sensors was also evaluated by recording CV voltammograms of 1.0 nM DMMP in the B-R buffer of pH 4. The peak current intensity (*i*_p_) voltammograms were recorded without applying adsorptive accumulation time (open circuit conditions) (*i*_pIII_), followed by utilizing an adsorptive accumulation time of 75 s (*i*_pI_ 1st cycle and *i*_pII_ 2nd cycle). According to the adsorptive step, a well-defined peak was noticed at the GCS_2_ (*i*_pI_; in Fig.S_8_(B)), owing to the great enhancement in the adsorption property toward DMMP even at open circuit conditions (*i*_pIII_). Moreover, the remarkable decline of the *i*_pII_ observed in the 2nd cycle may be due to the desorption of DMMP from the sensor surface. Based on the last-mentioned findings, we can deduce that GCS_2_ has the best affinity toward the determination of DMMP, which could be used in electroanalytical studies due to its good catalytic, adsorptive, and selective properties.

#### Optimization of the pH medium and analytical parameters

Because of the ion exchange process between the detected sample and the surface of sensor, the *pH* of solution generally has a significant impact on the *i*_p_ signal and the clarity of voltammograms of the detected sample on the surface of as-prepared sensors. Thus, the effect of changing pH values of B-R buffer (2–8) as a supporting electrolyte on the *i*_p_ signal of 0.7 nM of DMMP was tested at *E*_acc_ =  − 1.5 V (vs. Ag/AgCl-KCl) for 30 s using GCS_2_ as displayed in Fig.S_9_. The oxidative *i*_p_ signal was strongly affected by changing the acidity of the solution from 2 to 7, followed by the complete disappearance of the *i*_p_ at pH ≥ 8. The highest oxidative peak was obtained at pH 4, which was subsequently utilized for optimizing instrumental operational conditions.

The optimum instrumental operational conditions for the detection of DMMP were pointed out to be pH 6, *E*_acc_ =  − 1.5 V, *t*_acc_ = 30 s, frequency (*f*) = 90 Hz, pulse amplitude (*a*) = 30 mV, and scan increment (Δ*E*_s_) = 10 mV using GCS_2_, as displayed in Figs.S_10_(A, B, and C). According to the adsorption step, the effect of changing *E*_acc_ from − 1.7 to − 1.3 V (vs. Ag/AgCl-KCl) on the *i*_p_ signal of 2.0 pM of DMMP in B-R buffer pH 4 was estimated for 30 s using GCS_2_, as displayed in Figs.S_11_(A) and (B).

Also, the effect of changing the *t*_acc_ of 0.3 and 0.7 nM of DMMP on the *i*_p_ signal was estimated, as displayed in Fig. S_11_(C). According to the last-mentioned results, *E*_acc_ =  − 1.5 V and *t*_acc_ at 30 s are the optimum accumulation conditions for the determination of DMMP in B-R buffer of pH 4, which are employed for the subsequent electroanalytical measurements.

### The electroanalytical detection of DMMP

#### Limits of quantification (LOQ) and detection (LOD)

**Fig. 4:**
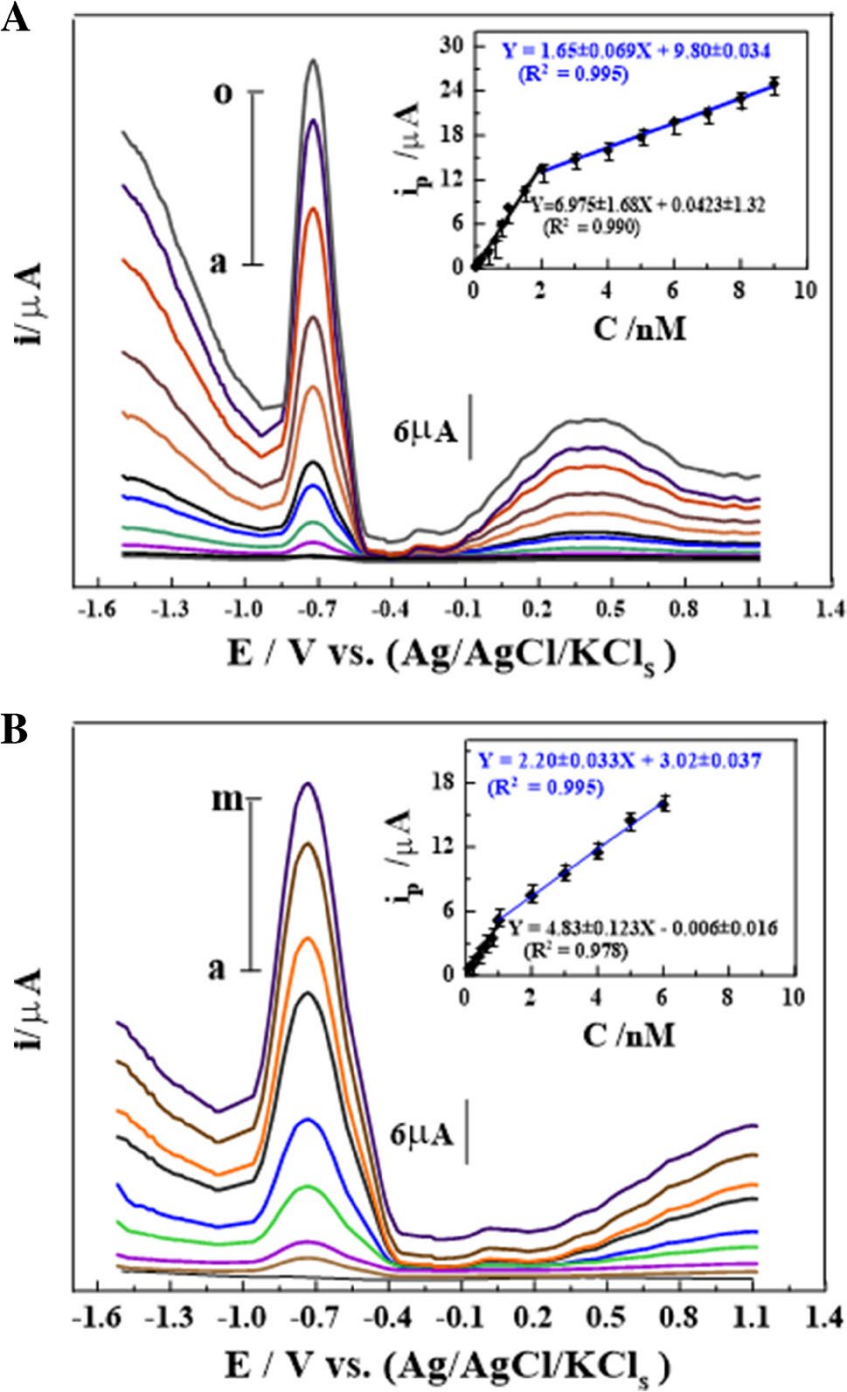
**A** SW-AdAV voltammograms of different amounts of DMMP in *pH* 4 on GCS_2_ (*E*_acc_ =  − 1.5 V, *t*_acc_ = 60 s, Δ*E*_s_ = 10 mV, *f* = 90 Hz, and *a* = 30 mV) in bulk form: (a) baseline, (b) 0.04, (c) 0.09, (d) 0.3, (e) 0.6, (f) 0.7, (g) 1.3, (h) 1.7, (m) 5.5, (n) 8.0, and (o) 9.0 nM (inset: its corresponding plot (*n* = 3)). **B** SW-AdAV voltammograms of different amounts of DMMP in *pH* 4 on GCS_2_ (*E*_acc_ =  − 1.5 V, *t*_acc_ = 60 s, Δ*E*_s_ = 10 mV, *f* = 90 Hz, and *a* = 30 mV) in spiked serum form: (a) baseline, (b) 0.1, (c) 0.2, (d) 0.6, (e) 1.0, (f) 3.0, (g) 4.0, (h) 5.0, and (m) 6.0 nM (inset: its corresponding plot (*n* = 3))

Under the chosen analytical conditions, the calibration curve of different concentrations of DMMP showed two linear portions over the ranges of 0.02–2.0 and 2.0–9.0 nM into the B-R buffer of pH 4 at the GCS_2_ was characterized by SW-AdASV, as displayed in Fig. 4A. The linear regression equations of the last-mentioned calibration plots were expressed as follows: *i*_p/μA_ = (6.975 ± 0.138) *C*_DMMP/nM_ + (0.0423 ± 0.0047) (*R*^2^ = 0.990), and *i*_p/μA_ = (1.650 ± 0.33) *C*_DMMP/nM_ + (9.8 ± 0.36) (*R*^2^ = 0.994) with a limit of detection (LOD) ≈ 0.06 pM (sensitivity of 6.975 μA/pM). As summarized in Table S1, different types of methods and sensors have been developed to detect DMMP in gaseous and liquid states. Among the mentioned findings in Table S1, we can conclude that GCS_2_ has the lowest LOD value and wider linearity range (LR) toward the detection of DMMP in bulk and biological fluid (serum sample) at room temperature (RT) compared to the other reported analytical methods to date. Furthermore, there are no detailed reports on the usage of the SW-AdASV technique for the detection of DMMP in bulk and biological fluids.

#### Validity of the as-modified sensor

Under optimum conditions, reliability and repeatability could be evaluated throughout the day (intra-day analysis) and every day over 3 days (inter-day analysis) by recording SW-AdAS voltammograms of 0.3 nM DMMP using five freshly as-modified GCS_2_ in parallel. The recovery ± relative standard deviation (R% ± RSD) of the intra-day and inter-day analysis of DMMP were 101.51% ± 3.28 and 99.3% ± 3.36, respectively, revealing the excellent reliability and repeatability of the as-modified sensor. Furthermore, the stability of the as-modified sensor has been checked three times (*n* = 3) every 7 days through its storage in air for 30 days at RT, maintaining 96.36% of its initial activity for more than 15 days and reaching 92.3% after 30 days, as figured out in Fig.S_12_(A). As tabulated in Table.S_2_, the results proved that the sensor exhibited proper reliability, repeatability, and long-term stability, owing to the chemical stability of the as-prepared Cd ZIF-67 modifier.

#### Selectivity

The anti-interference performance (selectivity) of the GCS_2_ was investigated by the addition of common interferences in human biological systems, as displayed in Fig.S_12_(B). The *i*_p_ voltammogram of 0.3 nM of DMMP (*C*_I_) (Fig. S_11_(B); a) was evaluated after addition of 30.0 nM (~ 100-fold) of common metal cations (Mix1: Ca^+2^, Mg^+2^, Fe^+2^, Zn^+2^, Co^+2^, Na^+^, and K^+^) and 30.0 nM (100-fold) mixture of S-containing amino acids (S-amino: cysteine (Cys) and thiamine (TA)), as demonstrated in Fig.S_12_(B); b. Noteworthy, there was no noticeable difference in the magnitude of the *i*_p_ voltammogram of 0.3 nM of DMMP with an RSD of 2.88%, and no additional peaks of Mix_1_ and S-amino interferents appeared. Moreover, Fig.S_12_(B); c exhibits a new peak at 0.36 V (*C*_II_), which corresponds to 0.03 nM (15-fold) uric acid (UA) with a relative error (RE %) ~  ± 1.2 at the DMMP peak. Furthermore, Fig.S_12_(B); d demonstrates another peak at 0.15 V (*C*_III_) after the addition of 30.0 nM (~ 100-fold) of the mixture of other interferents (Mix_2_: ascorbic acid (AA), dopamine (DA), and glucose (Glu.)).

On the other hand, the specificity performance of the GCS_2_ was investigated toward the detection of DMMP in the presence of compounds with similar electroactive functional groups such as glyphosate (GLYP) and chlorpyrifos (CPYP) in bulk form, as displayed in Scheme.S_1_ and Fig.S_13_. The *i*_p_ voltammogram of 0.6 nM of DMMP (Fig. S_12_; a) was evaluated with the same value even after the addition of 60.0 nM of GLYP, which demonstrated a small peak at − 0.02 V, corresponding to (GLYP). According to previous reports by Wu et al. [44], Indra et al. [45], and Liu et al. [46], the limitation in the detection response of GLYP during the detection of DMMP could arise from the usage of an optimized *pH* medium (universal buffer; *pH* 4), which is far from the reported optimized pH condition (phosphate buffer; *pH* 7) in the detection of GLYP. Moreover, the *i*_p_ voltammogram of 0.6 nM of DMMP did not show any additional oxidation peak associated with the addition of 60.0 nM of CPYP as a result of it not being oxidized (Fig. S_13_; b) [47]. These results proved that the presence of common interferences in human biological systems and other compounds with the same electroactive functional groups does not interfere with the detection of DMMP, even when the concentrations exceed 100-fold.

### Application in spiked human serum fluid

The GCS_2_ was applied to detect DMMP, spiked in human serum samples of 3 healthy volunteers in the B-R buffer of *pH* 4 using SW-AdASV under the chosen analytical conditions without the necessity for sample pre-treatment steps. SW-AdAS voltammograms of different concentrations of DMMP in the presence of spiked human serum samples (Voulanter_1_) over two linear ranges of 0.1–1.0 and 1.0–6.0 nM into the B-R buffer of *pH* 4 at the GCS_2_ with a LOD ≈ 0.03 nM (sensitivity of 4.83 μA/nM), as displayed in Fig. 4B. Moreover, 0.6 and 1.0 nM of DMMP were estimated in the presence of spiked human serum samples (Voulanter_2, 3_), which achieved a good recovery (R %) ± relative standard deviation (RSD %) without interference from other biological contents, as summarized in Table.S_3_. These results proved that the fabricated sensor exhibited proper accuracy (RE %) and reliability in the detection of DMMP even among complex biological systems.

## Conclusion

This work involved the successful hydrothermal synthesis of porous nanoparticles of Cd ZIF-67 with a rhombic dodecahedral morphology, which were then applied to the construction of an electrochemical sensor for DMMP. The porous Cd ZIF-67 nanoparticles possessed a high surface area, while 1.0% of the modified GCS (GCS_2_) promoted the electrocatalytic activity and adsorption behaviors of BGCS. Subsequently, the GCS_2_ exhibited low limit of detection in bulk with proper reliability, long-term stability, and repeatability, which werenecessary for the electroanalytical detection. Furthermore, the fabricated modified sensor exhibited a proper accuracy and reliability in the detection of DMMP even among complex biological systems.

### Supplementary Information

Below is the link to the electronic supplementary material.

Supplementary file1 (DOCX 1327 KB)

## Data Availability

Data available on request from the authors.
