# Supplementary material for: Adsorptive stripping voltammetric sensor based on Cd zeolitic imidazole framework-67 for electrochemical detection of sarin simulant
Source: Mikrochim Acta. 2024 Jan 8;191(2):80. doi: 10.1007/s00604-023-06112-3 (PMC10774163; doi:10.1007/s00604-023-06112-3)
Supplement: Supplementary file 1 — Supplementary file1 (DOCX 1327 KB) [file 604_2023_6112_MOESM1_ESM.docx]

**Electronic Supporting Material**

**Adsorptive stripping voltametric sensor based on Cd zeolitic imidazole framework-67 for electrochemical detection of sarin simulant**

**Mona Elfiky^1*^, Amr M. Beltagi^2^, Osama Abuzalat^3*^**

*^1^Department of Chemistry, Faculty of science, Tanta University, Tanta, Egypt*

*^2^Department of Chemistry, Faculty of Science, Kafrelsheikh University, 33516 Kafrelsheikh, Egypt*

*^3^Department of Chemical Engineering, Military Technical College, Cairo, Egypt*

**Corresponding author e-mail:* [*Elfiky_mona@science.tanta.eu.eg*](mailto:Elfiky_mona@science.tanta.eu.eg&osama.abuzalat@mtc.edu.eg)*,* [*Osama.abuzalat@mtc.edu.eg*](mailto:Osama.abuzalat@mtc.edu.eg)

*
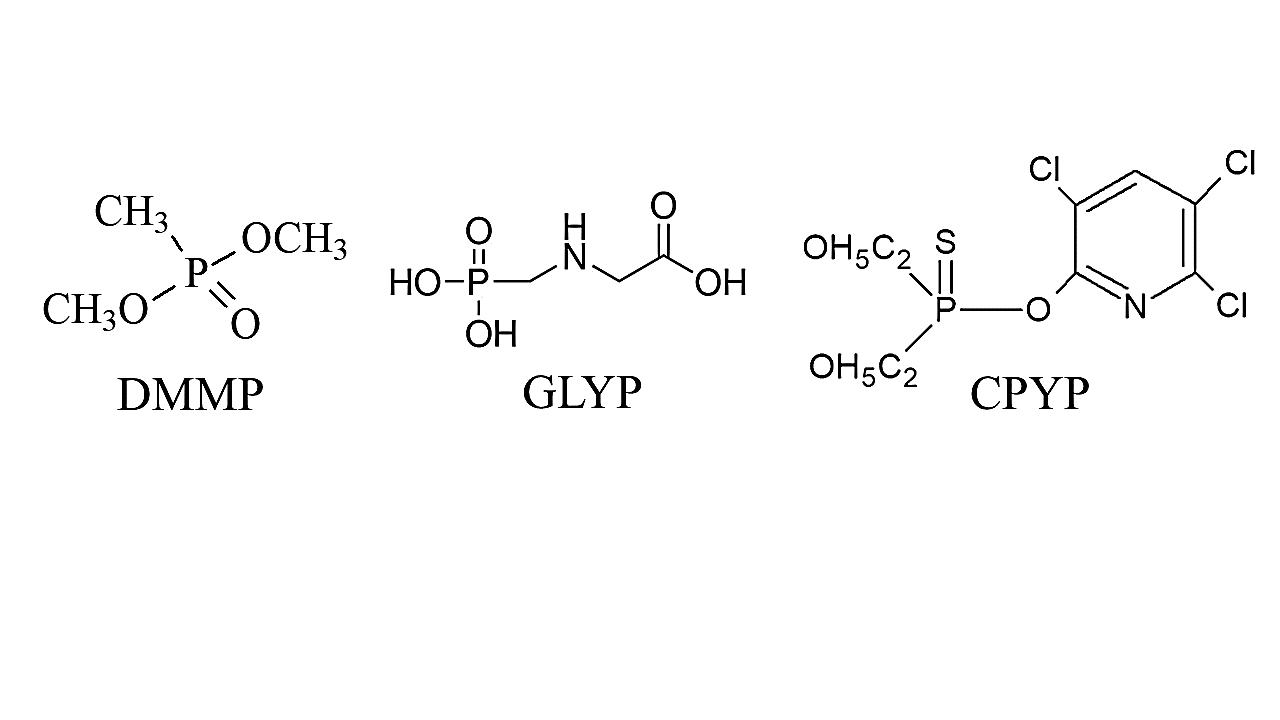
*

**Scheme S_1_.**Structure of dimethyl methyl phosphonate (DMMP), glyphosate (GLYP), and chlorpyrifos (CPYP).

**Table. S_1_.**Numerous analytical methods for the detection of DMMP.

| **Material** | **Method/ Type of sensor** | **Processing temp. /^o^C** | **LOD or LR** | **Refs.** |
| --- | --- | --- | --- | --- |
| Al doped (1.0 %) ZnO NPs | [Hydrothermal](https://www.sciencedirect.com/topics/chemistry/hydrothermal-method)/ gas sensor | 350 | 0.8 µM (100 ppb) | [**^4^**](#_ENREF_4) |
| Mo5Sb1-SnO_2_ | [Hydrothermal](https://www.sciencedirect.com/topics/chemistry/hydrothermal-method)/gas sensor | 350 | 0.81 µM (0.1 ppm) | [**^5^**](#_ENREF_5) |
| SnO_2_NPs | [Hydrothermal](https://www.sciencedirect.com/topics/chemistry/hydrothermal-method)/ gas sensor | 350 | 0.81 µM (0.1 ppm) | [**^6^**](#_ENREF_6) |
| ZnO (4 wt %) doped SnO_2_ | [Hydrothermal](https://www.sciencedirect.com/topics/chemistry/hydrothermal-method)/ gas sensor | 250-400 | 4.03 µM (500 ppb) | [**^7^**](#_ENREF_7) |
| Ni-doped SnO_2_ | [Hydrothermal](https://www.sciencedirect.com/topics/chemistry/hydrothermal-method)/ gas sensor | 500 | 40.3 µM (5000 ppb) | [**^8^**](#_ENREF_8) |
| CuO NPs/ZnO | [Hydrothermal](https://www.sciencedirect.com/topics/chemistry/hydrothermal-method)/ gas sensor | 300-400 | 1.5–30 mg/m^3^ | [**^9^**](#_ENREF_9) |
| NiFeAl^3+^/NFls | Gas sensor | - | 0.8 µM (0.1 ppm) | [**^10^**](#_ENREF_10) |
| Ppy/ AuS | CV | RT | - | [**^11^**](#_ENREF_11) |
| QCL-PAS | LPAS | RT | 5.6 nM (0.7 ppb) | [**^12^**](#_ENREF_12) |
| WS_2_ [thin films](https://www.sciencedirect.com/topics/materials-science/thin-films) coated QCM | Chemi-resistive gas sensor | RT | 0.04 µM (5 ppb) | [**^13^**](#_ENREF_13) |
| HFIPP-GR film-modified QCM | Chemi-resistive gas sensor | RT | 1.2 µM (150 ppb) | [**^14^**](#_ENREF_14) |
| HFM-SPME | CD-IMS | RT | (4.03 – 230.6 nM)  0.5–50 µg mL^−1^ | [**^15^**](#_ENREF_15) |
| PS cartridge | PS-MS | RT | 0.1 µM (12.6 ng mL^−1^) (Urine)  0.23 µM (28.6 ng mL^−1^) (Blood) | [**^16^**](#_ENREF_16) |
| SiNWFET Sensor | Gas sensor | RT | 0.81 µM (100 ppb) Bulk (Vapor) | [^17^](#_ENREF_17) |
| GCS_2_ | SW–AdASV | RT | 0.06 pM (Bulk)  0.03 nM (Serum) | **This work** |

# Nanoparticle (NPs), Polypyrrole/ gold sensor (Ppy/ AuS), quantum cascade laser photoacoustic spectroscopy (QCL-PAS), coated [quartz crystal](https://www.sciencedirect.com/topics/physics-and-astronomy/quartz-crystal) microbalance (QCM) [oscillators](https://www.sciencedirect.com/topics/physics-and-astronomy/oscillator), *p*-hexafluoro-isopropanol phenyl (HFIPP) functionalized graphene (GR), hollow fibre membrane-protected solid phase microextraction (HFM-SPME), corona discharge ion mobility spectrometry (CD-IMS), Aluminium-doped nickel ferrite (NiFeAl^3+^) nanoflakes (NFls), laser photoacoustic spectroscopy (LPAS), paper spray mass spectrometry (PS-MS), Silicon Nanowire (SiNW) FET Sensor.

**Experimental part**

**1. Materials, apparatus, electroanalytical solutions, and the point of zero charges (pH_ZPC_) measurements**

**1.1.****Materials, apparatus, and electroanalytical solutions**

Cadmium nitrate tetrahydrate (99.0 %, Sigma-Aldrich) and cobalt nitrate hexahydrate (98.0 %, Sigma-Aldrich), were used as metal ion sources. 2-methylimidazole (99.0 %, Sigma-Aldrich) was used as an organic ligand and triethylamine (99.0 % reagent grade, Fisher Scientific) were used as a deprotonating agent. Methanol (99.8%, Alfa Aesar) was used as a solvent for Cd ZIF-67powder synthesis. Dimethyl methyl phosphonate (DMMP, 99.8%, Alfa Aesar),[potassium hexacyanoferrate (III)](https://www.sigmaaldrich.com/EG/en/substance/potassiumhexacyanoferrateiii3292413746662) (K_3_[Fe(CN_6_)], ACS reagent, 99.0 %), phosphoric acid (H_3_PO_3_, 99.0%), boric acid (H_3_BO_3_, 99.5%), glacial acetic acid (CH_3_COOH, 99.0%), sodium chloride (NaCl, [ACS reagent, 99.0%](https://www.sigmaaldrich.com/EG/en/product/sigald/s9888)), sodium acetate (CH_3_COONa, [99.0%](https://www.sigmaaldrich.com/EG/en/product/sigald/s9888)), sodium carbonate (Na_2_CO_3_,  [99.5%](https://www.sigmaaldrich.com/EG/en/product/sigald/s9888),ACS reagent),[sodium phosphate monobasic](https://www.sigmaaldrich.com/EG/en/substance/sodiumphosphatemonobasic119987558807)(NaH_2_PO_4_,  [99.0%](https://www.sigmaaldrich.com/EG/en/product/sigald/s9888),ACS reagent), disodium hydrogen phosphate dihydrate (Na_2_HPO_4_.2H_2_O, 99.5%,EMSURE^®^), Sodium phosphate (Na_3_PO_4_, 96.0%), sodium sulphate (NaSO_4_, [99.0%](https://www.sigmaaldrich.com/EG/en/product/sigald/s9888),ACS reagent), sodium hydroxide (NaOH pellets, 98.0%), potassium chloride (KCl, 99.0%, ACS reagent) were all ordered from Merck, and used in the electroanalytical measurements.

A field emission scanning electron microscope (FE-SEM, QuantaTM 250) is used to inspect and analyze the morphologies of the Cd ZIF-67. An X-ray diffractometer (XRD, Rigaku Multiflex) is used to characterize the crystalline nature of the Cd ZIF-67. Fourier Transform Infrared Spectroscopy (FTIR) instrument (Nicolet Nexus 470, USA) is used to measure the transmittance spectra in the range of 4000−500 cm^-1^ with a spectral resolution of 4 cm^-1^.TheBrunauer-Emmett-Teller (BET)measurements are performed using a surface area analyzer (NOVA 3000e, USA) to determine thesurface areas of Cd ZIF-67. Raman spectra with laser excitation at 785 nm were performed using an alpha300 R Confocal Raman Microscope (WITec GmbH). A 100X objective microscope was used to focus the laser beam on the sample to a size of ~300 nm (Diffraction Limit by 532 nm). Information about the delocalization of charge throughout the synthetic molecule was obtained from the calculated Mulliken charge. The calculation was carried out using Gaussian 09 program package. Concerning the density functional theory (DFT) method using the functional Becke 3-Lee-Yang-Parr (B3LYP) in conjunction with standard 6-311 + G(d,p) basis sets.Electrochemical measurements were made using computer-controlled PAR (Princeton Applied Research, Oak Ridge, TN, USA).

A stock solution of dimethyl methyl phosphonate (DMMP) (1 mM) was freshly prepared and diluted in the range of (0.001–100 µM) in double deionized water (DDW). A series of Britton–Robinson (B-R universal) buffer (***pH*** 2–11) were prepared by adding different ratios of 0.04 M of (H_3_BO_3_, H_3_PO_4_ and CH_3_COOH), which adjusted to the desired pH with 0.2 M NaOH in DDW and used as a supporting electrolyte. A stock solution of K_3_[Fe(CN_6_)] (1mM) and 0.1 M of KCl were freshly prepared as a redox probe cell system and used in the electrochemical studies of the as-prepared sensor. Serum samples of three healthy volunteers were used to study the applicability of the developed sensor for the determination of DMMP spiked in human serum. The three volunteers gave their written informed consent before participating in the study and were authorized by the Ethics Committee for Research Involving Human Beings (Ref. no. KFSIRB200-7). Samples of the human serum (each of 1.0 mL) were spiked with the desired concentrations of DMMP in small centrifugation tubes (3.0 mL polypropylene microcentrifuge tubes). Each of these samples was then completed to 2.0 mL with ethanol (as a proteins precipitating agent). After vortexing each of the serum samples for 2 min, the precipitated proteins were separated by centrifugation for 3 min at 14,000 rpm. The clear supernatant layer was filtered through 0.45 μm Millipore filter to obtain protein-free spiked human serum samples with different concentrations of DMMP.

**1.2. The point of zero charges (*pH*_ZPC_) measurements**

The point of zero charges (***pH*_ZPC_**) of the Cd ZIF-67 powder was checked by the ***pH*** drift method [[28](file:///F:\After%20PhD\full%20papers\8-%20Dr.%20Osama\Paper%20II\Final\paper%2010-9-2022%20(Final)(sensors).docx#_ENREF_28), [29](file:///F:\After%20PhD\full%20papers\8-%20Dr.%20Osama\Paper%20II\Final\paper%2010-9-2022%20(Final)(sensors).docx#_ENREF_29)]. Briefly, a series of 0.01 M NaNO_3_ (20 mL) solutions (***pH*_i_** values) was adjusted in the range of 2.0 to 10 by using 0.1 M HNO_3_ or NaOH. Afterward, 0.06 g of Cd ZIF-67 powder was inserted into each adjusted NaNO_3_ solution with continuous stirring for 48 h. The ***pH*** of the final solution (***pH*_f_**value) was recorded. Then, the ***pH*_ZPC_** of the Cd ZIF-67was evaluated from the plot of **δ*pH*** (***pH*_f_**− ***pH*_i_**) vs.***pH*_i_**.

**1.3. Electrochemical measurements**

The electrochemical measurements were carried out in a micro-electrochemical cell with a three-electrode system, including modified GCS as the working sensor, Ag/AgCl/KCls as the reference electrode, and platinum wire as the auxiliary electrode. The electroactive surface area of all prepared GCSs was investigated in 10.0 mM K_3_[Fe(CN_6_)] in 0.1 M of KCl by CV technique, the potential was applied in the range of -0.4 and 1.1 V with a **v** of 100 mV s^−1^. The (SW-AdASV) technique scans were carried out using the chosen sensor in a micro-electrochemical cell (10 mL volume) containing a chosen amount of DMMP, DW and filled with a specific ***pH*** value under chosen accumulation conditions. SW-AdASV voltammograms were obtained after applying the +ve potential range.


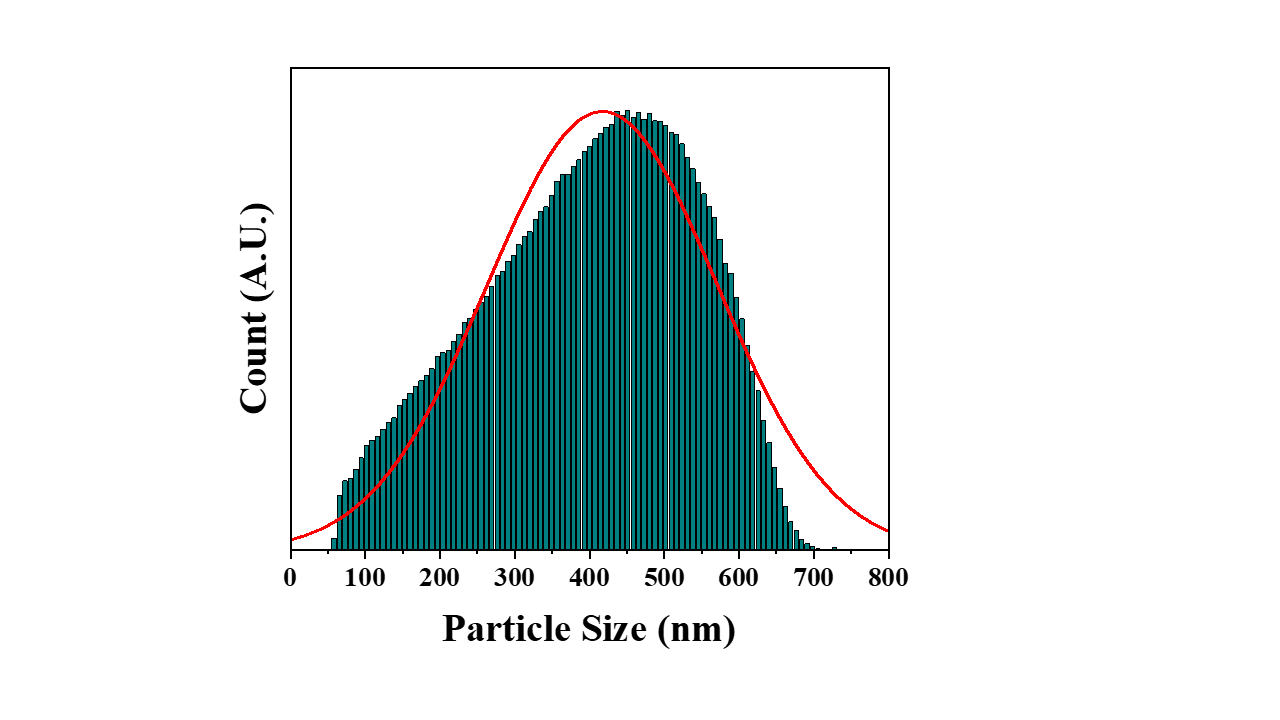


**Figure.S_1_.** The size distribution curve of Cd ZIF-67 material.


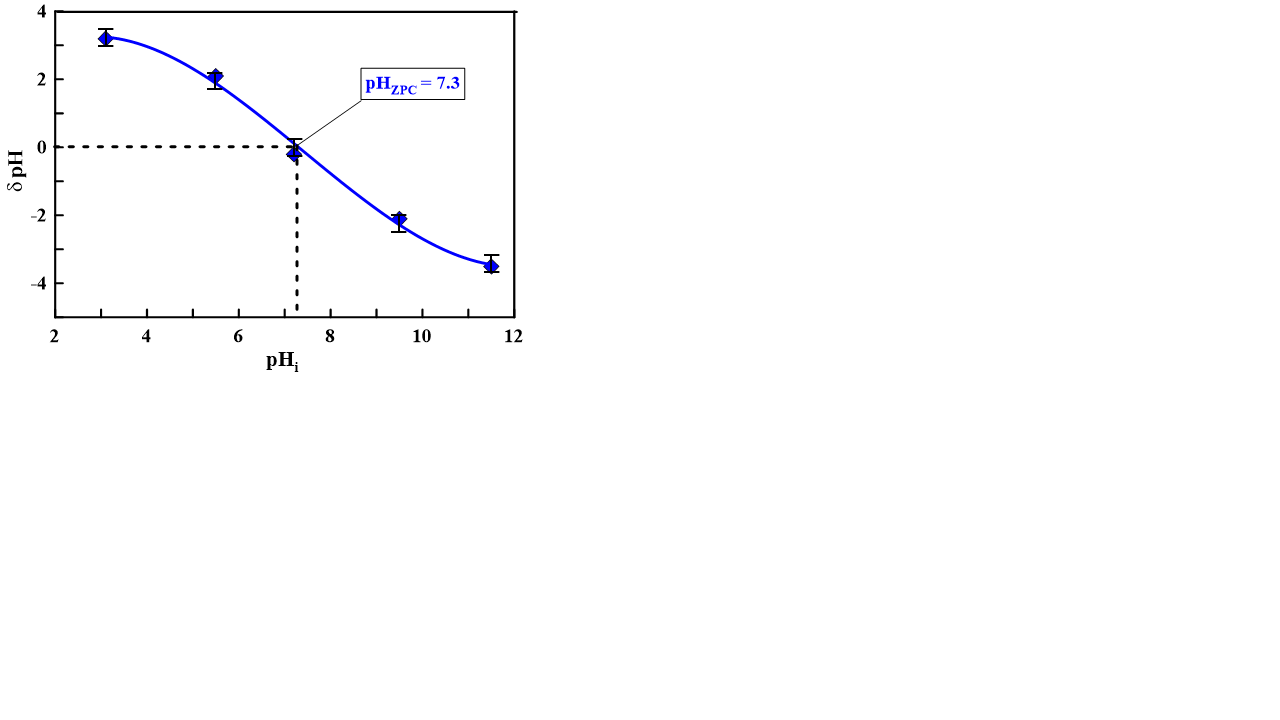


**Figure.S_2_.** The plot of ***δpH*** (***pH*_f_**-***pH*_i_**) vs. ***pH*_i_** including the value of ***pH*_ZPC_** of Cd ZIF-67 material.


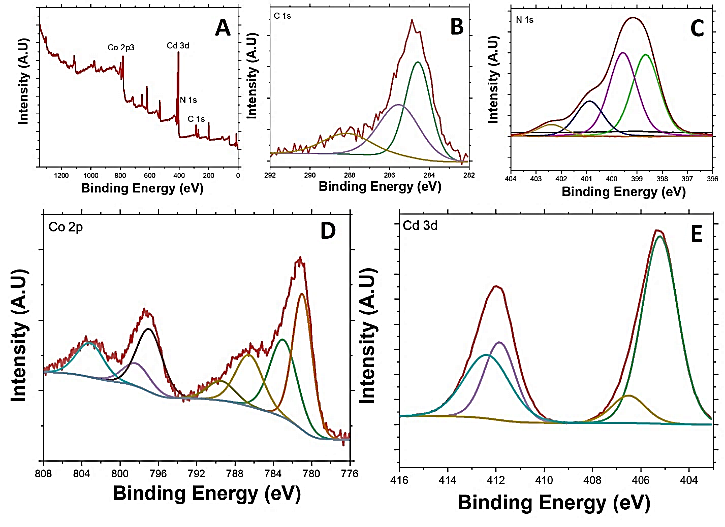


**Figure S_3_.** XPS analysis of Cd ZIF-67, (A) XPS survey spectra, (B) C 1s peaks, (C) N 1s peaks, (D) Co 2p peaks, and (E) Cd 3d peaks.

**Figure. S_4_**. CV voltammograms of (a) BGCS (red line), and (b) GCS_2_ (blue line) in a 0.5 M HCl solution at a scan rate of 100 mV·s^−1^.

**
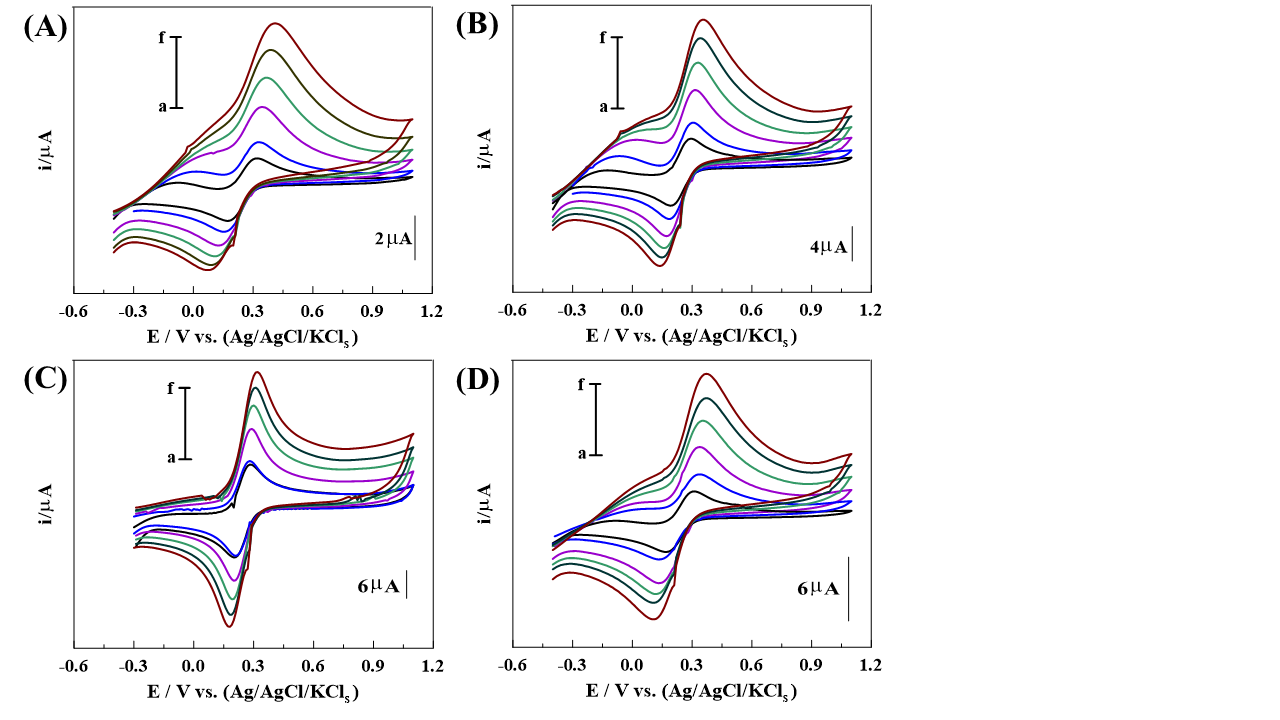
**

**Figure. S_5_.** CV voltammograms of 1.0 mM of K_3_[Fe(CN_6_)] in 0.1 M of KClat***v*** ≈ 20- 400 mV·s^−1^ using (A) BGCS, (B) GCS_1_, (C) GCS_2_, and (D) GCS_3_ sensors (***n***= 3).

**Figure. S_6_.** CV voltammogramsof (a) baseline, and (b) 1.0 μM DMMPin ***pH***4 using GCS_2_ at ***v***= 100 mV·s^−1^.


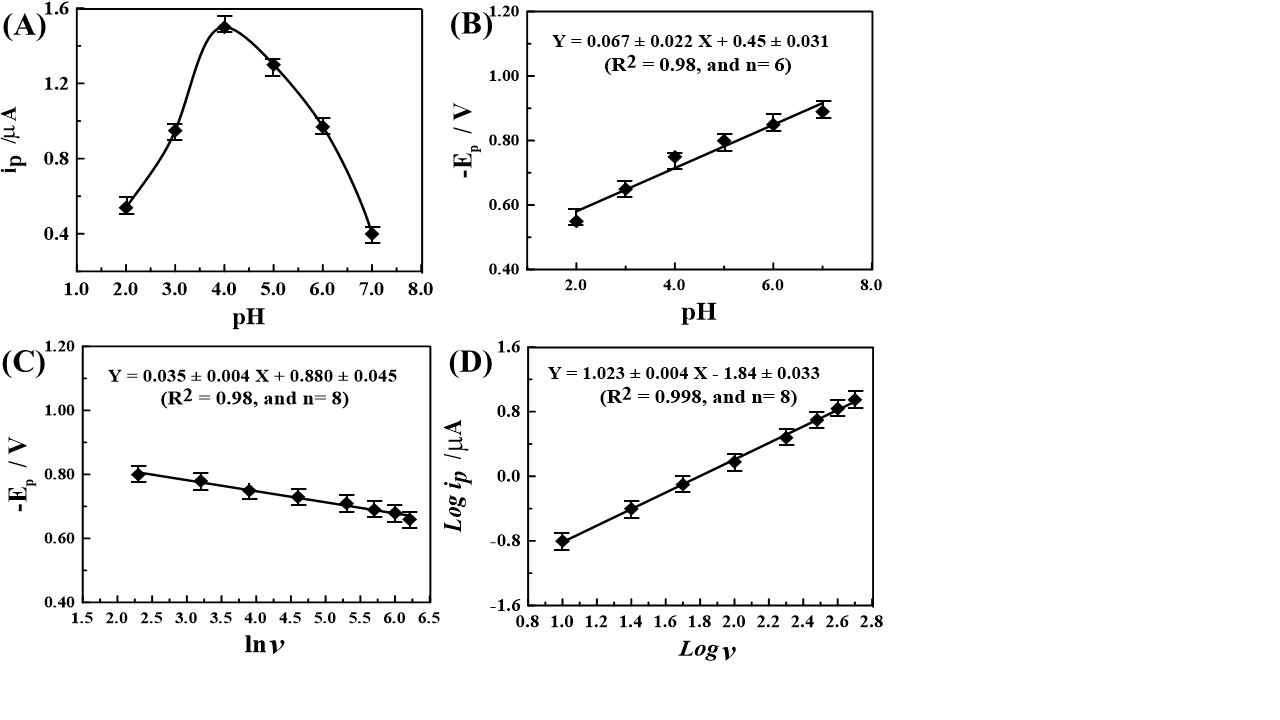


**Figure. S_7_.** CV voltammograms plots of **(A)*i*_p_** vs. ***pH***, and **(B) *E*_p_** vs. ***pH*** of 1.0 μM DMMP in different B-R universal ***pH*** values using GCS_2_ at ***v***= 100 mV·s^−1^. CV voltamograms plots of **(C) *E*_p_** vs. ln ***v***, and **(D)** Log ***i*_p_** vs. Log ***v*** in***pH*** 4 using GCS_2_ at ***v***≈ 100 mV·s^−1^.


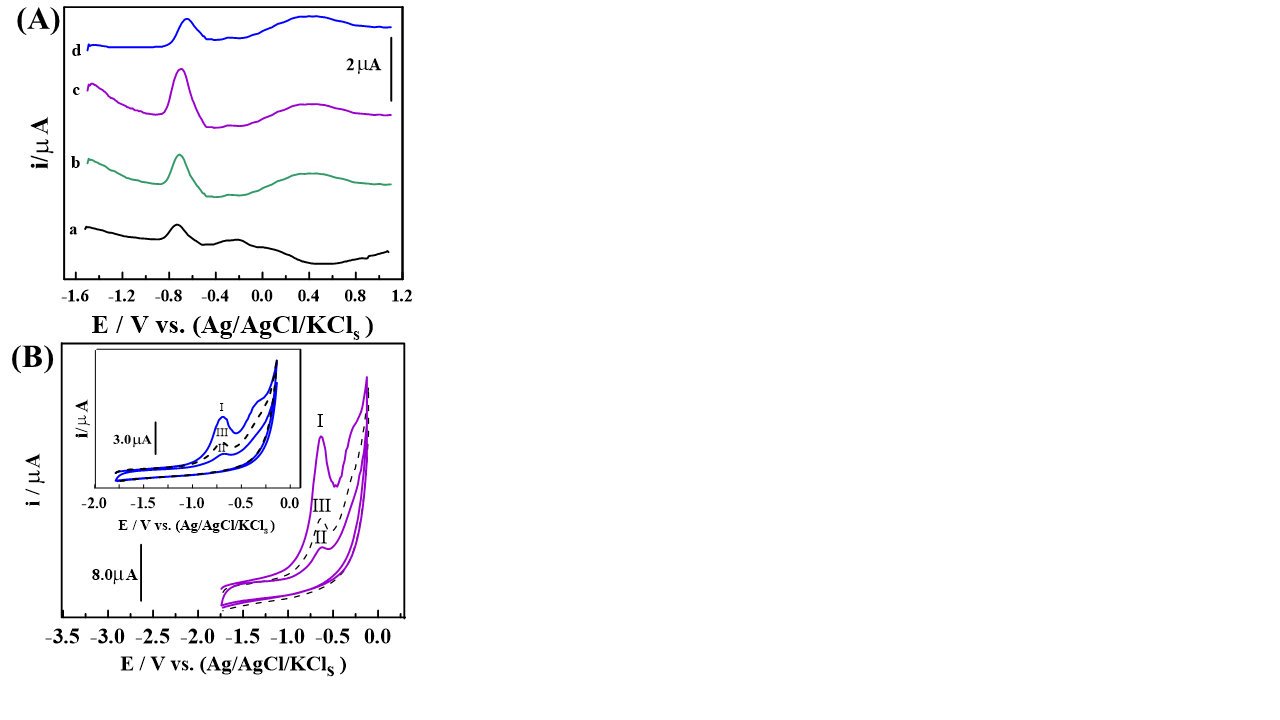


**Figure. S_8_**. **(A)** SW-AdASV peaks of 0.7nM of DMMP in the B-R universal buffer***pH*** 4 utilizing (a) BGCS, (b) GCS_1_, (c) GCS_2_, and (e) GCS_3_ sensors (***t_acc_***= 30 s, ***E_acc_***= −1.5 V, ***a***= 25 mV, ***f***= 100 H_z_, and **Δ*E_s_***= 10 mV). **(B)** CV voltammograms of 1.0nM of DMMP in the B-R buffer ***pH*** 4 BGCS (inset), and GCS_2_ at ***E*_acc_**=-1.5 V for ***t_acc_***= 30 s; (1^st^ cycle (I), and 2^nd^ cycle (II)), and under open-circuit conditions (III) ***v***=100 mV s^−1^.

**Figure. S_9_.**SW-AdASV voltammograms of different ***pH*** values (B-R universal buffer)of 0.7nMDMMP at ***E*_acc_**= -1.5 V (vs. Ag/AgCl-KCl) for 30 s using GCS_2_.

**
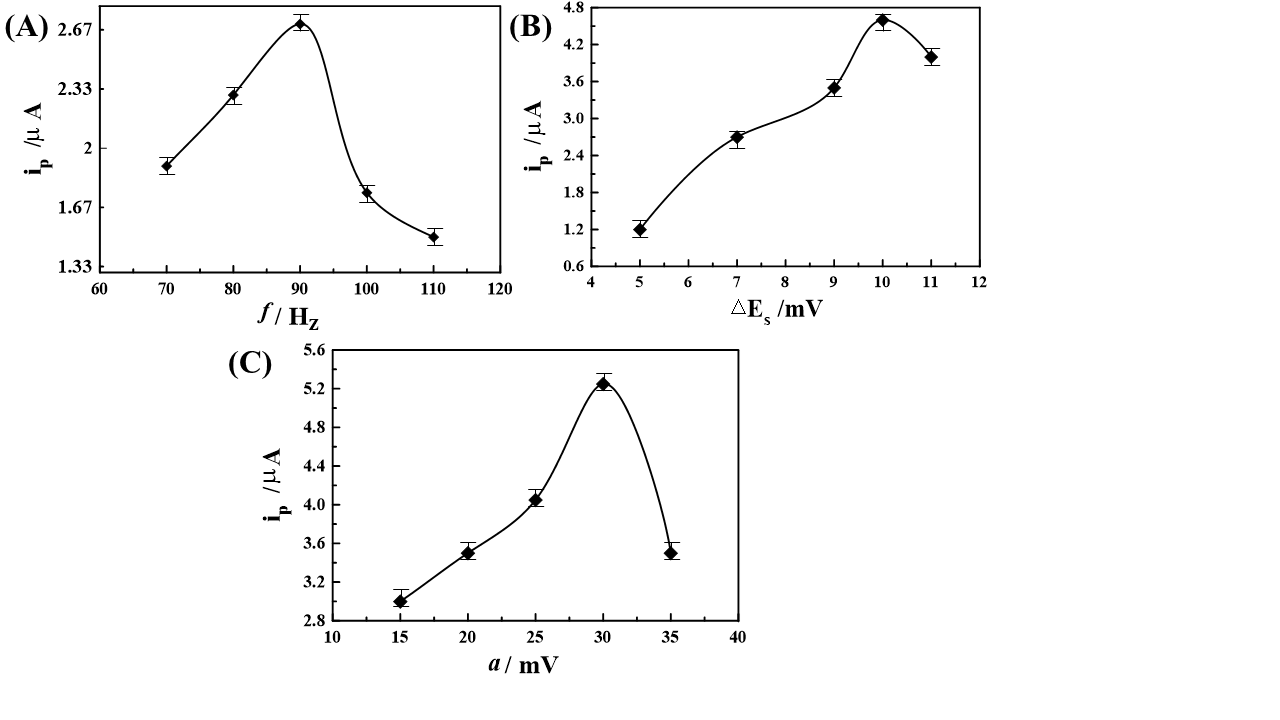
**

**Figure. S_10_.**SW-AdASVplots of ***i*_p_**vs.**(A)**frequency (***f***), **(B)** pulse height *(****∆E***_s_), and **(C)**scan increment (***a***)of 0.7nMDMMP at ***E*_acc_**= -1.5 V for 30 s using GCS_2_.


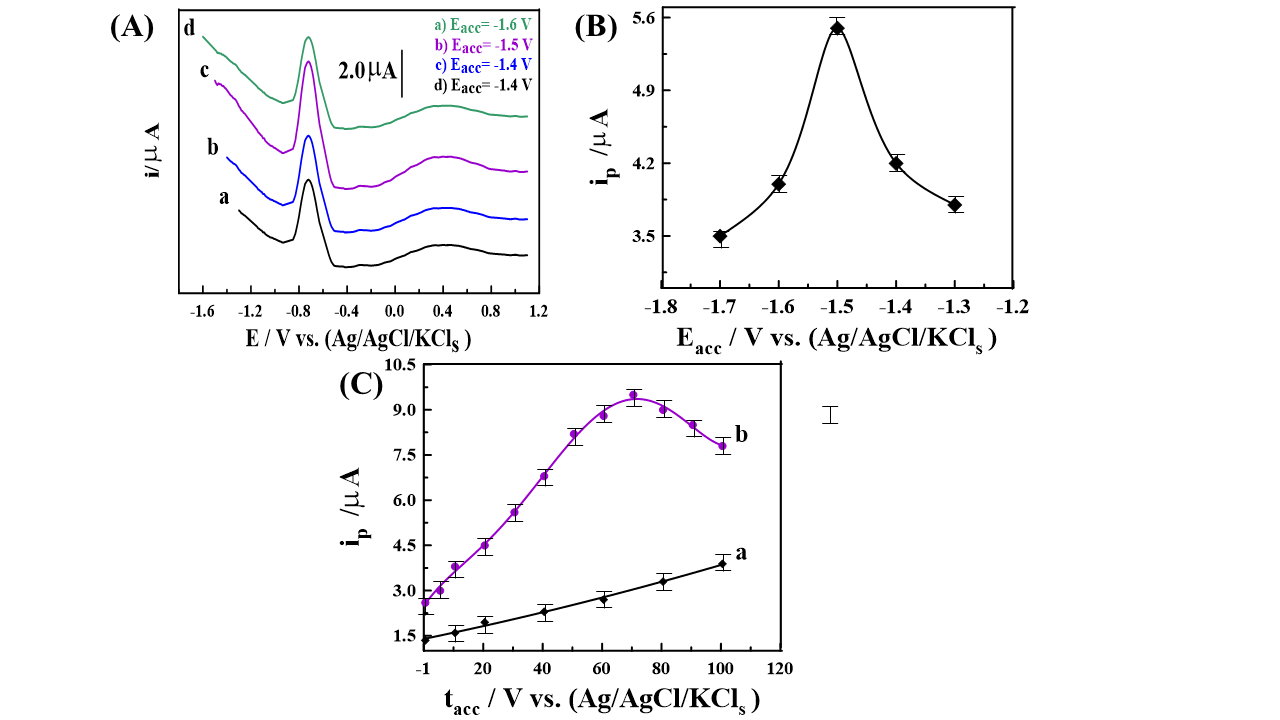


**Figure. S_11_. (A)**The effect of changing ***E*_acc_**on the SW-AdASV***i_p_***signal of 0.7 nM of DMMP in the B-R buffer of ***pH*** 4 at the GCS_2_ (***t_acc_***=30 s, ***a***=30 mV, ***f***=90 H_z_, and **Δ*E_s_***=10 mV), and **(B)** its corresponding histogram. **(C)** The effect of changing ***t_acc_*** on the SW-AdASV ***i_p_*** signal of (a) 0.3, and (b) 0.7nMon the SW-AdASV***i_p_***signal of 0.7 nM of DMMP in the B-R universalbuffer of ***pH*** 4 at the GCS_2_ (***E*_acc_**= -1.5 V, ***a***= 30 mV, ***f*** = 90 H_z_, and **Δ*E_s_*** =10 mV).

**Table. S_2_.**The intra- and inter-day analysis of 0.3nM DMMP in bulk using the SW-AdASV technique:

|  | ***C*_Taken_(nM)** | ***C*_Found_(nM) ± SD** | **Recovery ± Precision (R % ± RSD %)** | **Relative Error E_r_ (%)** |
| --- | --- | --- | --- | --- |
| ***Intra-day analysis*** | 0.3 | 0.305 ± 0.01 | 101.51 % ± 3.28 | 1.51 |
| ***Inter-day analysis*** | 0.3 | 0.298 ± 0.063 | 99.3 % ± 3.36 | -0.80 |
| ***Stability _(n = 3)_****  ***15 days***  ***30 days*** | 0.3 | 0.289 ± 0.004  0.277 ± 0.007 | 6.36 ± 1.38  92.3 ± 2.53 | -3.64  -7.70 |

* (***n***= 3) means three times of measurements.


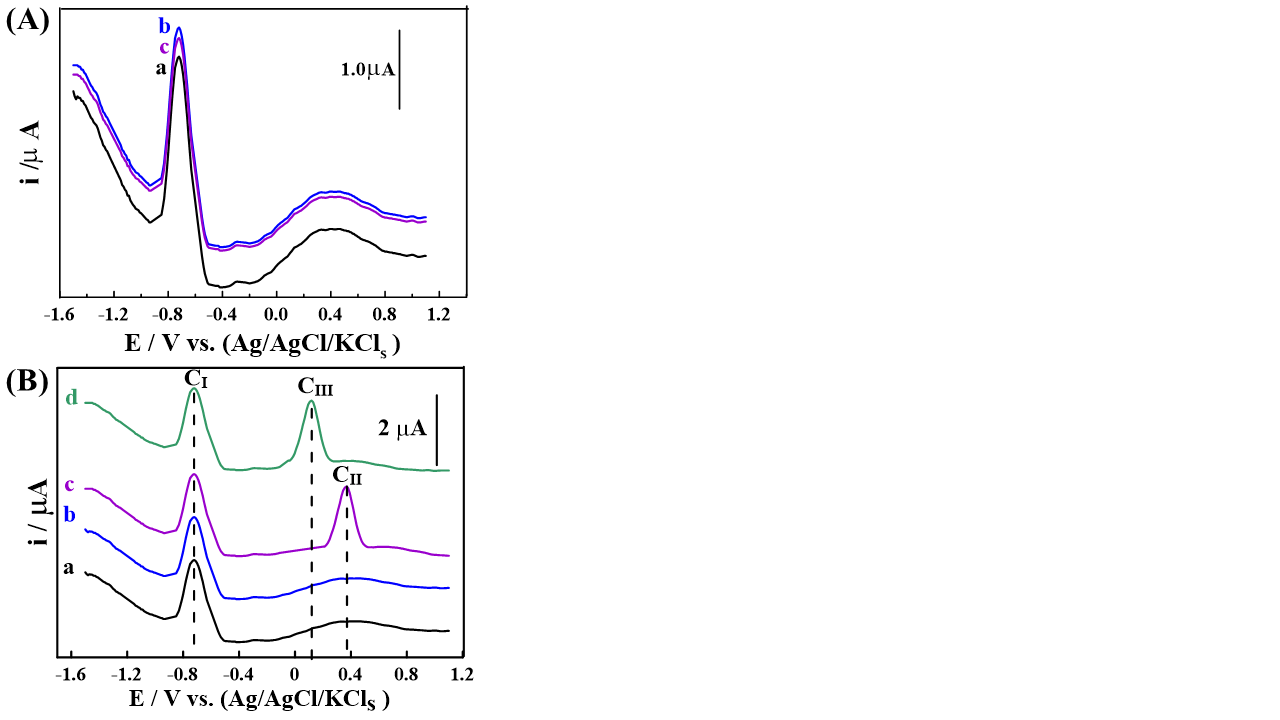


**Figure. S_12_. (A)** SW-AdAV voltammograms for 0.3 nM of DMMP(***pH*** 4) **(a)** initial check, **(b)** after 15 days, and **(c)** 30 days on the surface of **GCS_2_**.**(B)** SW–AdAV voltammograms for **(a)** 0.3 nM DMMPmixed with **Mix_1_**, **(b)**0.3 nM DMMP mixed with **Mix_1_**, and S-amino, **(c)** 0.3 nM DMMP mixed withUA, and **(d)** 0.3 nM DMMP mixed with **Mix_2_** on the surface of**GCS_2_**.

**Figure. S_13_.** SW–AdAV voltammograms for 0.6 nM of DMMP (***pH*** 4) in the presence of **(a)** 60.0 nM of glyphosate (GLYP), and **(b)** 60.0 nM of chlorpyrifos (CPYR).

**Table. S_3_.**Detection of DMMP in human serum samples (*n* = 3)

| **Sample** | **C_Added_(nM)** | **C_Found_(nM) ± SD** | **R % ± RSD%** | | **RE%** |
| --- | --- | --- | --- | --- | --- |
| ***Volunteer_2_*** | 0.10  0.60  1.0  3.0 | 0.101± 0.005  0.610± 0.021  0.973± 0.036  3.032± 0.06 | | 101.00 ± 5.19  101.66 ± 3.46  97.30 ± 3.84  101.0 ± 1.89 | 1.00  1.66  -2.70  1.00 |
| ***Volunteer_3_*** | 0.1  0.60  1.0  3.0 | 0.102± 0.005  0.615± 0.024  0.961± 0.015  3.022± 0.07 | | 102.00 ± 4.53  102.5 ± 3.86  96.10 ± 1.60  100.73 ± 2.19 | 2.00  2.50  -3.90  0.73 |
